# Clinical spectrum of lamellar macular defects including pseudoholes and pseudocysts defined by optical coherence tomography

**DOI:** 10.1136/bjo.2007.133041

**Published:** 2008-08-06

**Authors:** J C Chen, L R Lee

**Affiliations:** 1 City Eye Centre, Brisbane, Australia; 2 Institute of Health and Biomedical Innovation, Queensland University of Technology, Brisbane, Australia; 3 Department of Ophthalmology, University of Queensland, Royal Brisbane Hospital, Brisbane, Australia

## Abstract

**Objective:**

To present the clinical spectrum of lamellar macular defects and describe the different subtypes based on their optical coherence tomography (OCT) configuration and visual prognosis.

**Methods:**

The retrospective observational case series reviewed OCT scans of 92 eyes with lamellar macular defects. Lamellar macular defects were categorised into subtypes of macular pseudohole (MPH), lamellar macular hole (LMH) and foveal pseudocyst (FP) according to their OCT morphology. The defects were quantitatively characterised in terms of base diameter, depth and central foveal thickness, and examined for the presence of associated epiretinal membranes (ERM).

**Results:**

Visual acuity (VA) was significantly correlated with the central foveal thickness and depth of the lamellar defect. MPH was associated with better VA compared with LMH and FP. MPH was of a smaller base diameter and had a greater central foveal thickness than that of LMH and FP. Fifty-per cent of all lamellar defects had an associated ERM.

**Conclusions:**

Different profiles of lamellar macular defects were characterised and quantified by OCT. Deeper and wider lamellar defects were associated with poorer visual outcome. Such objective parameters lamellar macular defects are of value when explaining to patients regarding their decreased acuity. Future prospective investigations are required to study the natural history of lamellar defects of different aetiology and surgical indications.

Optical coherence tomography (OCT) is an imaging modality that has revolutionised the way a myriad of ocular conditions are now diagnosed and monitored.^1^^–^8 OCT provides a cross-sectional tomography of the ocular microstructure in vivo and in real time, and the unprecedented high axial resolution (10 μm for standard OCT; 3–5 μm for ultra-high-resolution OCT) enables OCT the capability to provide information that cannot be obtained with any other ophthalmic diagnostic techniques on the internal architecture of retinal pathology.^1^ In particular, direct visualisation of the vitreomacular interface with OCT imaging has significantly enhanced the understanding of the tractional forces causing structural changes to the retinal anatomy, as well as providing insight into the underlying aetiology and pathogenesis of the vitreomacular interface abnormalities, including macular hole,^9^^–^12 lamellar hole,^13^ ^14^ macular pseudohole,^14^ epiretinal membrane^15^ ^16^ and vitreomacular traction syndrome.^13^ ^17^

Lamellar macular hole was first described by Gass in 1975 as a macular lesion that resulted from cystoid macular oedema.^18^ Since then, there has been much interest in defining the condition from other similar, yet distinct macular disorders, such as full-thickness macular holes, macular pseudoholes and vitreomacular traction.^9^ ^11^ ^13^ A lamellar macular hole is by definition a partial thickness macular hole where the inner layers of the fovea are involved with traction and detached from the underlying cellular layers of the fovea. Lamellar macular holes typically appear as a round or irregular-shaped, well-circumscribed reddish lesion on biomicropscopy, but clinical detection of lamellar holes at an early stage can be difficult. Several studies have found that lamellar holes are underdiagnosed when conventional techniques are used.^13^ ^14^ Only 28% (eight of 29 eyes) of lamellar holes diagnosed on OCT examination were detected clinically on fundus examination in the study of Haouchine *et al*,^14^ and 37% (seven of 19 eyes) in the study by Witkin *et al*^13^ With an OCT investigation, lamellar macular holes are easily diagnosed, and their characteristic features of an irregular foveal contour, break in the inner fovea, intraretinal split and an absence of a full-thickness foveal defect with intact foveal photoreceptors have been defined as a criterion for diagnosis.^13^

In addition to facilitating the diagnosis of lamellar macular holes, OCT has also been used to enhance the understanding of the aetiology and pathogenesis of the condition. Haouchine *et al*^11^ have identified foveal pseudocyst as a precursor to the development of both lamellar and macular hole formation. Lamellar holes are thought to be caused by de-roofing of the pseudocyst with preservation of the foveal base, while posterior extension of the pseudocyst leads to the formation of full-thickness macular holes.^11^

There have been very few OCT reports on lamellar macular holes, and our understanding of the definition, anatomical features, pathogenesis and surgical indications of this macular condition is limited. The purpose of this paper was to present the varied presentations of lamellar macular defects and describe features characterised by OCT that may be correlated to visual prognosis and the need for surgical intervention.

## MATERIALS AND METHODS

We retrospectively reviewed the clinical records of 92 eyes of 81 consecutive patients (23 males and 58 females) who were referred to our centre for investigation of lamellar macular defects including holes, pseudoholes or pseudocysts between January 2004 and July 2007. The mean age of the patients was 67.7 (SD 9.4) years (range 39–87 years). The study was conducted in accordance with the tenets of the Declaration of Helsinki and the requirements of the Queensland University of Technology Human Research Ethics Committee. Informed verbal consent was obtained from all patients after explanation of the nature of the study.

A complete ocular examination was performed including best corrected visual acuity (VA) measurement, slit-lamp biomicroscopy and fundus photography. VA was measured on a Snellen chart and converted to the logMAR notation for statistical analysis. All eyes with macular defects were evaluated with OCT 3 (Carl Zeiss Meditec, Dublin, CA). OCT is based on an optical technique known as Michelson low-coherence interferometry, which measures the echo delay and intensity of back-reflected or backscattered infrared light (∼800 nm) to produce high-resolution, cross-sectional tomography of ocular strucutres.^19^ OCT 3 provides a maximum of 512 (transverse)×1024 (axial) data points per image, acquired in 1.92 s, and has an axial resolution of approximately 10 μm. All images were captured using the Macular Thickness Map protocol where six radial scans of 6 mm length, at equally spaced angular orientations centred on the macula, were performed. Images were then processed using the Retinal Thickness/Volume protocol, and the software caliper was used to obtain parameters on lamellar macular defects (see below).

### Subtypes of lamellar macular defects

Lamellar macular defects were categorised into three different subtypes based on their OCT appearance: macular pseudoholes (MPH), lamellar macular holes (LMH) and foveal pseudocysts (FP). Lamellar macular defects which had a sharply punched out, well-delineated, steepened foveal contour were classified as MPH. OCT profiles corresponding to LMH had a thin and irregular foveal floor overlying the plane of the retinal pigment epithelium. In many cases of LMH, the central defect also extended laterally, resulting in a split between the inner and outer retinal layers. FP appeared as a cystoid space occupying mostly the inner part of the fovea, with a perifoveal detachment of the posterior hyaloid. In some cases of FP, there was an elevation of the foveal floor, which suggests vitreomacular traction from adherence of the partially detached posterior hyaloid to the roof of the FP.^11^

### OCT measurements of base diameter, depth and retinal thickness

**Figure 1:**
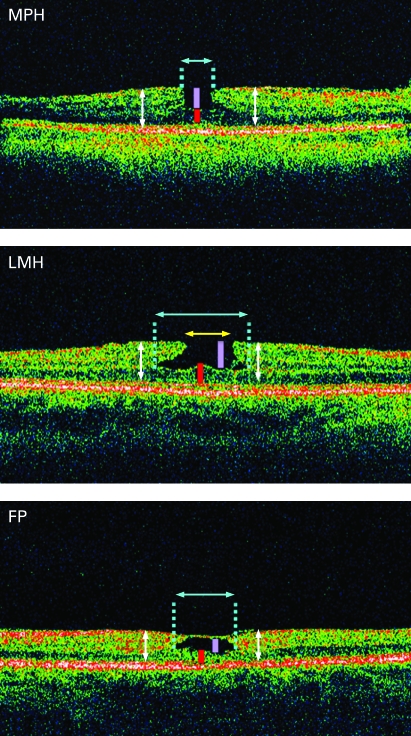
Optical coherence tomography scans of lamellar macular defect measurements showing placement of calipers for base diameter, depth, central foveal and perifoveal retinal thickness measurement for the three types of defects: macular pseudohole (MPH), lamellar macular hole (LMH) and foveal pseudocyst (FP). For LMH, the opening (minimum) diameter of the lamellar hole was also measured. The blue arrows indicate the base diameter of the defects, while the yellow arrow indicates the opening diameter. The purple bar indicates the depth of the lamellar defect; the red bar indicates the central foveal thickness; and the white arrows indicate retinal thickness at points 750 μm either side of the foveal centre. The perifoveal retinal thickness was calculated as the average of the two measurements on either side from the foveal centre.

Measurements of base diameters and depth of lamellar macular defects were obtained using the software calipers. As lamellar macular defects were frequently asymmetrical and irregular, macular profiles of the lamellar defects varied depending on the orientation of the scan. All six radial scans were examined to find the scan which revealed the deepest and widest defect from which all measurements were obtained. Figure 1 shows examples of parameter measurements for the three types of lamellar macular defect on OCT scans. Measurements of base diameters and depths were made to the most lateral extent of the intraretinal split and the thinnest part of the foveal base respectively. Both the base diameter and the opening (minimum) diameter were measured for LMH. While FP defects were not open macular defects and had a roof over the cystic area, the measured depth here referred to depth of the cystic space. Retinal thickness was measured at the foveal centre (central foveal thickness) and at points 750 μm from either side of the foveal centre. The two measurements from both sides of the foveal centre were averaged and referred to as the perifoveal retinal thickness. The presence of epiretinal membrane was also noted for each lamellar macular defect.

### Statistical analysis

Statistical analysis of the data was conducted using the Statistical Packages for the Social Sciences (SPSS 14.0.1). One-way analysis of variance (ANOVA) was used to determine if there were any differences in OCT parameters for MPH, LMH and FP. In all cases where comparisons between MPH, LMH and FP were made, a Bonferroni-corrected post hoc test was used, adjusting the observed significance level for multiple comparisons. Correlations were also performed to establish if there was any relationship between OCT parameters and VA.

## RESULTS

**Figure 2:**
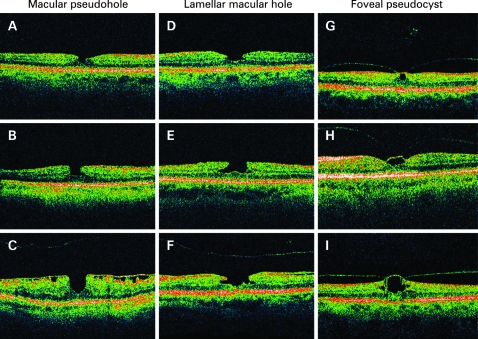
(A) Optical coherence tomography (OCT) scan of a sharply punched-out defect corresponding to a macular pseudohole (MPH). (B) OCT scan of an MPH of larger diameter compared with (A) and verticalisation of the foveal contour. (C) As the MPH became deeper, it also assumed a more oval shape. A thick epiretinal membrane bridging deep retinal folds was observed. (D) OCT profile of a lamellar macular hole (LMH) with small lateral cleft. (E) LMH, of larger diameter compared with (D) with lateral intraretinal split, assuming a “bilobate” contour. (F) Extensive lateral intraretinal split between the inner and outer retinal layers and irregular foveal base of the LMH. Note the detached posterior hyaloid. (G) Foveal pseudocysts (FP), which appeared as an intraretinal cystoid space occupying the inner part of the foveola. Partially detached posterior hyaloid was also evident. (H) Posterior hyaloid, which remained adherent to the foveal centre, exhibiting a biconvex linear signal and elevating the roof of the pseudocyst, suggesting vitreomacular traction. (I) Increased cystoid spaces on the edge of the cyst laterally between the inner and outer retinal layers, as the size of the FP increased.

OCT scans of the 92 eyes were analysed and categorised into three different subgroups of lamellar macular defects: MPH, LMH and FP. Thirty-nine eyes had OCT features of well-defined, punched out defects corresponding to MPH; 26 eyes were in the LMH subgroup, and 27 eyes had FP. Figure 2 shows the varied presentations of the three types of lamellar macular defects, with examples of each subtype showing varying diameters and depths.

The VA of the 92 eyes with lamellar macular defects at initial presentation ranged from 20/15 to 20/200. The VA differed significantly between the three subtypes of lamellar macular defects (F_2,89_ = 8.272, p = 0.001). The MPH group had the best VA of 0.1 (0.15) logMAR (equivalent to 20/25 Snellen acuity) compared with that of the LMH group (0.28 (0.25) logMAR; p = 0.005) and the FP group (0.30 (0.26) logMAR; p = 0.002) (table 1). The VA of LMH and FP was similar (p = 1.00).

**Table 1:** Optical coherence tomography (OCT) parameters of the three different types of lamellar macular defects

Types of lamellar macular defect	MPH (n = 39) (SD)	LMH (n = 26) (SD)	FP (n = 27) (SD)	F and p Value*
Initial VA (logMAR)	0.10 (0.15)†‡	0.28 (0.25)	0.30 (0.26)	F_2,89_ = 8.272, p = 0.001
Base diameter (μm)	452 (178)†‡	1022 (632)§	707 (377)	F_2,89_ = 15.184, p<0.0001
Defect depth (μm)	182 (54)†‡	216 (54)§	144 (47)	F_2,89_ = 12.757, p<0.0001
Central foveal thickness (μm)	141 (26)‡	122 (36)	104 (34)	F_2,89_ = 10.747, p<0.0001
Perifoveal thickness (μm)	322 (54)‡	338 (57)‡	281 (22)	F_2,89_ = 10.488, p<0.0001

*One-way analysis of variance.

†Statistical significance between MPH and LMH, post-hoc Bonferroni analysis, p<0.05.

‡Statistical significance between MPH and FP, post-hoc Bonferroni analysis, p<0.05.

§Statistical significance between LMH and FP, post-hoc Bonferroni analysis, p<0.05.

FP, foveal pseudocyst; LMH, lamellar macular hole; MPH, macular pseudohole; VA, visual acuity.

The difference in central foveal thickness of the three subtypes of lamellar macular defects followed the same pattern as the difference observed in VA (F_2,89_ = 10.747, p<0.0001; table 1). MPH had the thickest central foveal thickness measurement (141 (26) μm), which was significantly greater than LMH (122 (36) μm, p = 0.047) and FP (104 (34) μm; p<0.0001). The differences in central foveal thickness of LMH and FP were not significantly different (p = 0.128). There was a statistically significant correlation between VA and central foveal thickness (r = −2.33, p = 0.025), that is, the thicker the central foveal thickness, the better the VA.

There were also significant differences in the base diameter of the lamellar defects for the three groups (F_2,89_ = 15.184, p<0.0001; table 1). LMH had the widest base diameter (1022 (632) μm), which was significantly greater than that of the MPH group (452 (178) μm; p<0.0001) and the FP subgroup (707 (377) μm, p = 0.019). The difference between MPH and FP was also statistically significant (p = 0.044). The opening (minimum) diameter of LMH was similar to the width of MPH (442 (173) μm, cf. 452 (178) μm; p = 1.00).

In addition to having defects of larger base diameters, LMH also had deeper defects compared with MPH and FP (F_2,89_ = 12.757, p<0.0001; table 1). The average depth of LMH was 216 (54) μm, which was significantly greater than that of MPH (182 (54) μm, p = 0.033) and FP (144 (47) μm; p<0.0001). The depth of MPH was significantly greater than FP (p = 0.014). There was also a statistically significant correlation between VA and depth of the lamellar macular defect (r = 0.222, p = 0.034), that is, the deeper the lamellar defect, the poorer the VA. Perifoveal thickness measurements of MPH and LMH were similar (322 (54) μm *cf.* 338 (57) μm; p = 0.593). The perifoveal thickness of FP (281 (22) μm) was significantly less than that of MPH and LMH (p = 0.002) (table 1).

**Figure 3:**
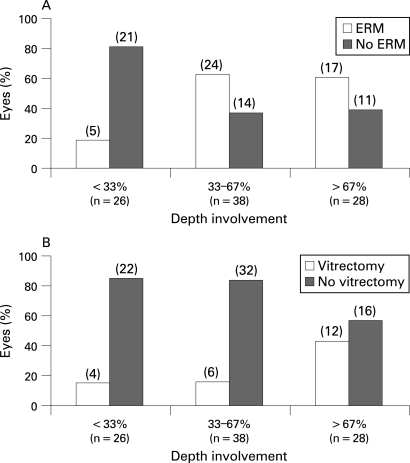
(A) Number of eyes with or without epiretinal membrane (ERM) based on the depth of lamellar macular defect, expressed as a percentage of normal perifoveal retinal thickness. As the depth of the lamellar macular defect increased, the number of eyes with ERM increased. For depth over 33% of retinal involvement, over 60% of defects had associated ERM. (B) Number of vitrecomised and non-vitrecomised eyes based on the depth of lamellar macular defect, expressed as a percentage of normal perifoveal retinal thickness. The majority of shallower defects (over 80%) did not undergo a vitrectomy, while close to half of lamellar defects with more than 67% of retinal thickness involvement underwent a vitrectomy.

Fifty per cent of all lamellar defects had associated ERM. The presence of ERM was associated with the depth of lamellar defects; lamellar defects with ERM had an average defect depth of 205 (61) μm compared with 156 (44) μm of defects without ERM (t_90_ = 4.417, p<0.0001). When the depth of the lamellar defect was expressed as a percentage of perifoveal thickness, over 60% of lamellar defects with greater than a third of retinal thickness involvement were associated with ERM (fig 3). This was significantly different to that observed for the shallower lamellar defects; less than 20% of lamellar defects with less than a third of retinal thickness depth involvement were associated with ERM (fig 3). The presence of ERM alone did not have any significant effect on VA (t_90_ = 0.670, p = 0.505).

## DISCUSSION

The varied presentation of lamellar macular defects presented in the study shows the wide clinical spectrum of macular disorders that may simulate a macular hole on clinical examination. Incorporating OCT imaging as part of the routine investigation for macular disorders not only makes differential diagnosis easier, but provides qualitative assessment and allows visualisation and classification of the different subtypes of lamellar macular defects, as well as quantitatively characterising the defects by providing objective measures such as base diameter and depth. OCT is also effective in evaluating the vitreoretinal interface to detect epiretinal membranes that are frequently associated with lamellar macular defects and implicated as a contributing factor in their formation.^13^

We have attempted in this study to provide correlations between OCT morphology of lamellar macular defects and visual function, which, to the best of our knowledge, has not been reported previously for lamellar holes. Among the different subtypes, we found that lamellar defects with a sharply punched out profile corresponding to MPH had the best level of acuity compared with that of LMH and FP. Morphologically on OCT scans, MPH had the smallest base diameter and thickest central foveal tissue, while LMH had the widest and deepest defects, and FP had the thinnest measured central foveal thickness. Our findings are consistent with the findings of Haouchine *et al*^14^ where MPH was found to be of a smaller diameter than that of LMH. The measured central foveal thickness in MPH (141 (26) μm) in our study was also comparable with the findings of Haouchine *et al*^14^ (167 (42) μm) and falls within the range of the normative data for central foveal thickness found by Massin *et al* (146 (20) μm).^20^

While the findings are consistent, there was one distinct difference between our study and that of Haouchine *et al*^14^ where we measured the widest intraretinal split as the base diameter rather than the opening of LMH. This in our opinion has allowed greater discrimination between the different subtypes of lamellar defects, as the opening diameter of LMH measured in our study was not significantly different from that of MPH. In addition, the characteristic lateral split between the inner and retinal layers has been suggested to be the result of the separation between the outer plexiform layer and the outer nuclear layer (ONL) or, more specifically, between Henle fibres and ONL.^21^ ^22^ If the photoreceptor axons are separated from the inner retina, then it could be suggested as one of the underlying causes of reduced vision in lamellar macular defects, in addition to the partial loss of foveal tissue. Measuring the intraretinal cleft therefore may correspond better to the reduction in vision and provide better differentiation from lamellar defects without the characteristic lateral split such as in MPH. In full-thickness macular holes, the associated visual loss is said to result from the direct loss of foveal tissue, and there is a suggestion that the thickness of cellular tissue between the base of the hole and the RPE layer is inversely proportional to the patient’s resulting VA.^1^ ^23^ It is thus reasonable to draw tentative conclusions from our findings that the higher level of acuity of MPH was attributable to the smaller diameter and thicker central foveal tissue, while the poor acuity of LMH and FP was due to the deeper/wider intraretinal split and thin foveal tissue respectively.

Throughout the manuscript, we have described MPH, LMH and FP under the same banner of lamellar macular defects, as they are all partial-thickness macular holes with preservation of some foveal tissue. They also all meet the OCT criteria of lamellar holes devised by Witkin *et al*,^13^ having an irregular foveal contour, a break in the inner fovea, an intraretinal split, and intact foveal photoreceptors. However, it has been suggested previously that MPH and LMH are entirely different entities.^14^ MPH is said to result from the centripetal contraction of an ERM that subsequently leads to verticalisation of the foveal slopes and a sharply punched out defect,^24^ whereas LMH has been suggested to result from an aborted process of full-thickness macular hole formation, with the characteristic split between the inner and outer retinal layers rather than a foveolar detachment.^11^ ^12^ ^23^ ^25^ FP has also been described as another separate entity as a precursor to macular hole or lamellar macular hole formation due to direct vitreomacular traction.^11^ While we have identified morphological features that are characteristic of each lamellar macular defect subtype, more studies are required to study the pathogenesis involved in the formation of lamellar macular defects.

As previously reported, epiretinal membranes are a common feature associated with lamellar holes, and they have been suggested to be involved in traction on the fovea and play a role in the formation lamellar holes. Fifty per cent of eyes with lamellar macular defects in our study had ERM, compared with 62% of 29 eyes in the study by Haouchine *et al*^14^ and 89% of 19 eyes in the study by Witkin *et al*.^13^ Related to the latter study, the higher proportion of ERM found in eyes with lamellar holes could be attributed to the increased resolution of ultrahigh-resolution OCT used.^13^ Measurements of perifoveal thickness of MPH and LMH were both outside the normal range of retinal thickness (233–253 μm),^20^ and it is likely that the presence of thickened ERM and retinal folds had confounded the measurements of perifoveal thickness in MPH and LMH. Perifoveal thickness of FP could have been thinner due to release of traction of the posterior hyaloid face. While ERM may have an important role to play in the pathogenesis of lamellar macular holes, it must be emphasised that it is not responsible for the formation of all types of lamellar macular defects discussed in this study. It is likely that while ERM may induce the formation of lamellar macular holes whereby ERM exerts a centripetal force on the fovea resulting in a cleft between the inner and outer retina, another mechanism involving avulsion of the foveal tissue or the roof of a pseudocyst can also lead to the formation of lamellar macular defects.^11^ ^14^ The natural history and visual prognosis of lamellar macular holes resulting from the two different mechanisms may potentially be different and should be studied in longitudinal studies using OCT.

A limitation of this study is that it was a retrospective review. Prospective and longitudinal studies involving a large number of lamellar macular hole cases would be useful to provide important information on the potential ability of OCT parameters to predict the natural history of lamellar macular defects, the likelihood of progression to full-thickness macular holes, as well as anatomical and functional outcome of surgical intervention.

In summary, the study presented the clinical spectrum of lamellar macular defects and demonstrated the use of OCT imaging in the classification and characterisation of different profiles of lamellar macular defects. Redefining lamellar macular defects into subgroups of macular pseudoholes, lamellar macular holes and foveal pseudocysts gave insight into their visual prognosis and underlying retinal morphology. Prospective long-term studies are important to broaden the understanding of the pathogenesis of each lamellar macular defect subtype and to evaluate the potential of OCT in predicting the natural history and surgical intervention of lamellar macular defects.
